# A Case of Sciatica Treated Successfully by Inoculation with Sulphate of Morphine

**Published:** 1851-09

**Authors:** Charles Brackett

**Affiliations:** Rochester, Ind.


ARTICLE III.

*A Case of Sciatica treated successfully by Inoculation with Sulphate of Morphine.* By CHARLES BRACKETT, M. D., of Rochester, Ind.

The following, if you think proper, you may publish. It is concerning a case of Sciatica, (I like the shorter, and full as expressive term, in lieu of the Neuralgia Femero-Poplitæ,) of long standing, which I treated by inoculating the skin over the course of the nerve with Sul. Morphine, made into a thin paste with Croton Oil.

This was a case of some years duration, and had been treated in this country and New York without an appearance of benefit.

The patient, Wm. R., aged about fifty years, of a spare habit but large and muscular frame, and active disposition, had suffered for the past ten or fifteen years with occasional rheumatic attacks, affecting generally his upper though often his lower extremeties and back. The pain, and weakness in his back, and in the course of the sciatic nerve for the past two years, had been persistent, so that he needed the aid of a cane when walking; for the past few months he had been confined to his bed, suffering such pain as only the victim ot Neuralgia has a knowledge of. I had tried most of the medicines which I thought could give him relief, both in the form of internal and external medication; at length I concluded to try this plan of inoculation, although I had not a remote idea of deriving *permanent* benefit from it, yet I could not bear the idea to give him up to the perpetual use of morphine, from which alone in large doses he found relief.

I began about the origin of the nerve, and inoculated the paste above mentioned about every four inches, down to his heel, which was as far as he felt any pain. That night he rested better than he had for a long time previously, the pain being entirely removed along the track of the inoculations; towards morning the pain attacked the Anterior Tibial Nerve, where previously it had never existed, and where it became as acute as ever it had been on the posterior part of his leg. I followed this pain up with my scarifications, putting in as much of the paste as I dared do in from four to six punctures made with the point of a thumb lancet at each place of inoculation. At this time I made my points of inoculation about three inches apart from the knee to the middle of the dorsal surface of the foot, so far as the pain existed; it ceased, and at my next visit it had appeared in the Plantar Nerves. I scarified and inoculated the sole of his foot, and from that time till his death he never suffered from any pain about that leg.

This patient, a robust Virginian, suffered more I think than any one I ever saw. Judging from his appearance, I thought it must be truly *perfect agony* he suffered.

He lived about a year after the cure of his Neuralgia, when he died from complicated disease of the Spleen and Liver, chronic in its character. As a *post mortem* was not allowed, I cannot give the exact condition of the viscera.

Though there is nothing remarkable about this case as respects the originality of its treatment, which I do not claim for it, yet the rapid and almost magical effects of the inoculation, together with the total and permanent disappearance of the neuralgic disease, I think probably ought to place it among the first remedies to be used in this disease, the treatment of which (through necessity from an absence of a knowledge of its existing causes) so often assumes an empirical tendency. At any rate it is to be considered a valuable adjuvant to other treatment.

